# Inter-operator Reliability for Measuring Pulse Wave Velocity and Augmentation Index

**DOI:** 10.3389/fcvm.2020.00072

**Published:** 2020-04-28

**Authors:** Catherine A. Elliot, Michael J. Hamlin, Catherine A. Lizamore

**Affiliations:** ^1^Department of Tourism, Sport and Society, Lincoln University, Christchurch, New Zealand; ^2^Department of Nursing, Midwifery and Allied Health, Ara Institute of Canterbury, Christchurch, New Zealand

**Keywords:** pulse wave velocity, augmentation index, pulse wave analysis, training, practice, experience, operator, SphygmoCor XCEL

## Abstract

**Background:** Arterial stiffness is a reversible precursor to hypertension. However, research is needed to determine the minimum amount of training required before acceptable arterial stiffness measurements are collected by novice operators.

**Objective:** To compare novice vs. experienced operator measurements over a 2-week training period to assess when expert-like measures are achieved by the novice operator.

**Method:** Forty-one participants (18 males, 23 females, age: 46.6 ± 14.9 years; BMI: 25.2 ± 3.8; systolic blood pressure: 122.8 ± 14.7 mmHg) received alternating novice and experienced operator arterial stiffness assessments. Measurements included: pulse wave velocity (PWV; using the automatic-capture time-periods of 5-, 10-, and 20-s) and augmentation index (AIx75) measurements using the SphygmoCor XCEL System v1 (AtCor Medical Pty Ltd., Sydney, Australia). Data were chronologically arranged into quintiles.

**Results:** The intraclass correlation coefficient for PWV substantially improved from quintile 1 (*r* < 0.8) to quintile 2 and beyond (typically *r* > 0.8) while AIx75 improved consistently (*r* = 0.7 in quintile 1 and r = 0.97 in quintile 5). The coefficient of variation was lowest in quintile 4 (PWV: 4.7–6% across the three measurement time-periods; and 15% for AIx75) but increased in quintile 5 (PWV: 6.2–10.5%; and 25% for AIx75). All measurements demonstrated acceptable to excellent reliability after quintile 2.

**Conclusion:** To achieve expert-like PWV measurements in this study, the novice operator underwent a familiarization session including guided practice measurements on 5 different people, for 10–15 min per person on two occasions (~2.5 h). The novice operator then required ≥14 practice measurements, with accuracy continuing to improve up to 30 participants. At least 30 training measurements are recommended for novices to take acceptable AIx75 measurements after a familiarization training.

## Introduction

Arterial stiffness is one of the earliest predictors of the onset of hypertension (1) and can be gauged by measuring pulse wave velocity (PWV) or pulse wave analysis (PWA). Pulse wave velocity is the gold standard in non-invasive arterial stiffness assessment (2), and is the transit time of a pulse wave measured between two pre-defined anatomical locations [distance traveled (m)/pulse transit time (s)]. In particular, the carotid-femoral PWV (cf-PWV) provides the most clinically relevant, non-invasive arterial health measurement as the pulse wave passes through the aortic artery (2).

Moreover, PWV is a strong predictor of future cardiovascular events (3), particularly in younger individuals at intermediate risk (4). Increased aortic PWV has been associated with reduced VO_2max_ performance in sedentary, middle aged participants (5), subclinical disease in coronary, lower extremity and cerebral arterial beds (6), and reduced skeletal muscle mass (7, 8). Furthermore, higher PWV has been associated with reduced cognitive ability in hemodialysis patients (9) as well as in older adults (10). The range in subjects receiving arterial stiffness assessments has also been broadened to include neonates (11), children (12, 13), adults (14), the elderly (15), and athletic populations (16). The wide use of these arterial stiffness measurements has enabled the establishment of normal and reference values aiding the assessment and interpretation of arterial health measures (17).

The central pressure waveform and its derived measures such as measurements of central pressure, pulse pressure and the Augmentation index (AIx) collected during the automated PWA provide an indication of peripheral arterial stiffness rather than central arterial stiffness as in PWV. AIx is the ratio of the central augmented pressure to the central pulse pressure expressed as a percentage and provides information on the interaction of the forward moving pressure wave (caused by ventricular contraction), with the backwards-moving reflective wave generated when the forward-moving wave meets a bifurcation in the artery (2). PWA has had broad application to numerous populations and in numerous contexts. For example, higher AIx has been associated with increased cardiovascular risk (18, 19), lower cardiorespiratory fitness (20), and increased clinical severity of coronary artery disease and percutaneous coronary intervention treatment in patients with a high Framingham risk score (18). In addition, higher AIx has also been associated with normotensive kidney disease (21), headaches and migraines in obese participants (22), and lower academic and motor performance in adolescents (23).

The rise in arterial stiffness research over the past few decades results from the availability of reasonably portable, relatively easy to use, time-efficient, and non-invasive devices. Recently, AtCor Medical Pty Ltd. released the SphygmoCor XCEL device which uses cuff-based volumetric displacement to detect the brachial and femoral pulse waves rather than applanation tonometry (24). These cuff-based measurements for PWA have vastly reduced measurement complexity and measurement time while providing validated methods of collecting arterial stiffness (25, 26).

Increased accessibility to more automated arterial stiffness measurement devices has meant that measuring arterial stiffness is no longer limited to a few highly-trained specialists. For example, some studies have reported measurements taken by relatively novice operators (27, 28). While these studies have reported good repeatability between novice operators, no comparisons were made between novice vs. experienced operators. To our knowledge, no evidence-based recommendations exist for the training periods required to achieve reliable expert-level measures of arterial stiffness. Therefore, there is a wide range in what constitutes a “well-trained” operator. For example, reported PWA training periods range from 2-day theoretical/practical workshops and 35 practice measurements (27), to 110 PWA measurements (29), while PWV measurements have been measured after as little as 15 practice measurements in work colleagues (28).

Subtle differences in tonometer technique, software literacy, and confidence of the operator may reduce measurement accuracy amongst novice operators. As Vlachopoulos et al. (30) found that a 1 m/s increase in PWV corresponded to an increase in cardiovascular risk by ~15% (after adjusting for age, sex, and risk factors). Any technique-related PWV measurement inaccuracy could have implications for clinical decision-making by health practitioners and the outcomes of care for their patients.

Regarding the SphygmoCor XCEL in particular, there are several measurement options in the module's capture settings. For example, if the operator selects the “automatic capture” option for their assessment of PWV, the operator may opt for an automatic capture period of 5, 10, or 20 s (as opposed to the operator selecting when to capture the pulse waves) wherein the software will capture recorded pulse waves over this duration, provided they are of sufficient quality. Aside from a recommendation to use the 20 s automatic capture for patients with unstable heart rates (31), very little advice is provided in the Operator's Manual regarding the best capture setting to use for consistent results. Reporting measurement capture duration settings is unconventional in arterial stiffness studies, thus little is known about which of these time-periods would provide the most reliable results when used by a novice operator.

Therefore, the first aim of this study was to monitor novice vs. experienced operator differences chronologically to determine the minimum training time required by novice SphygmoCor XCEL operators to reach expert-level accuracy. The second aim was to compare the differences between the inter-operator variability between 5, 10, and 20 s measurement capture times to provide a recommendation on which capture period is most reliable during the learning process.

In this study, the cf-PWV was measured using carotid applanation tonometry. Although it is not affected by wave reflection, it requires skill by the operator to obtain accurate applanation (flattening of the arterial wall) which can be difficult since the artery can move freely under the sensor and needs to be stabilized by pressure on nearby neck structures (32). PWA was measured using volumetric cuff displacement of the peripheral pressure waveforms to generate a corresponding central aortic pressure waveform (31, 33). One might assume that there would be more inter-operator variability of cf-PWV which requires accurate tonometer placement and pressure, as opposed to the automated measure of AIx which only requires proper cuff placement.

## Materials and Methods

### Participants

Participants were recruited from posters at a university's recreation center and through word-of-mouth. Inclusion criteria was participants ≥18 years of age who were able to attend a scheduled appointment in the morning. Exclusion criteria was participants who had a double mastectomy, were hypotensive or had or were treated for any cardiovascular disease (CVD) or peripheral artery disease (PAD). Hypotensives were excluded since they have a harder pulse to transduce, and are one of the more challenging patient groups for novice operators. Including hypotensive participants would give a distinct advantage to the more experienced operator, thus unduly impacting the inter-operator differences. Participants with CVD or PAD were excluded as some may have irregularities in blood pressure which could change for each operator, again, impacting inter-operator differences.

Forty-five participants were initially enrolled in the study, but four participants were later excluded from the dataset due to technical error (novice operator failed to move inflation hose from arm cuff to thigh cuff) (*n* = 2), environmental disruption causing disruption of measurement (*n* = 1) and the inability to capture PWV data by both operators (*n* = 1) due to high adipose tissue in neck (details in recommendations and conclusion).

The final dataset comprised 41 participants (18 males, 23 females, age: 46.6 ± 14.9 years; weight: 74.9 ± 14.8 kg; height: 171.9 ± 7.8 cm; BMI: 25.2 ± 3.8; systolic blood pressure: 122.8 ± 14.7 mmHg; diastolic blood pressure: 76.0 ± 8.9 mmHg; resting heart rate: 57 ± 9.2 bpm). Seventy-eight percent of the participants considered themselves physically active (≥150 min/week), with the remaining 22% physically active for <150 min/week. Ethical approval was obtained from the local university's Human Ethics Committee, and all participants provided written informed consent.

### Procedures

To understand the differences between an experienced and novice operator, 2 exercise-scientists collected data by serving as the device operators for this research project. The first operator had ~1 year experience with regular intervals of collecting PWV and AIx measurements using the SphygmoCor XCEL, testing over 80 people in total. The second operator had no experience collecting arterial health measures prior to this study. The novice operator was asked to read the entire operator's manual for SphygmoCor XCEL System v1 to familiarize herself with the device, measurement protocol and to become familiar with the software. The novice operator received 1-h of measurement training which comprised of technical information as well as PWA and PWV measurement demonstrations provided by the experienced operator and other trained operators from the research team. The novice operator then practiced on five different people, for 10–15 min per person on two occasions (2 just after the technical information and demonstration, and 3 on the morning prior to the research-related data collection). During these two training sessions, the experienced operator as well as two other trained operators from the research team provided feedback to the novice operator during and after testing and they answered her questions as needed. To ensure comparable familiarization prior to the examination of the test participants, the experienced operator also attended the technical information session, and performed similar “practice” measurements on the same preparation participants for 10–15 min each.

The novice and experienced operators alternated in their testing order whereby the operator who was first to collect data from the first participant would then be second to collect from the second participant and so on. Operators were not privy to each other's measurements and results. Each operator was allocated a maximum of 30 min to capture all the recordings which were conducted in the following order: height and body composition analysis, PWA, 5-s PWV, 10-s PWV, and 20-s PWV. Between operator recordings, the participant was asked to stand up and walk around for a few minutes to mimic the starting test conditions for the second operator as the participant walked to the first test from the building's reception.

The second operator then re-measured for height and body composition. All testing sessions followed identical protocols to reduce or eliminate any protocol-related measurement errors between operators. Pulse wave velocity and AIx data from each operator were recorded in an Excel spreadsheet. This grouping allowed for the comparison of measurement agreement between the novice and experienced operator over time to determine the effect of operator experience on measurement accuracy.

### Arterial Stiffness Assessment

Pulse wave velocity and PWA are two of the most commonly reported arterial stiffness measurements (34), and therefore comprised the focal measurements of arterial stiffness in the present study. Participant conditions were standardized according to the recommendations listed by the expert consensus document on arterial stiffness (2).

All measurements were conducted between 5:30 and 11:30 a.m. from Monday to Friday for 2 consecutive weeks. Participants were instructed to avoid strenuous physical activity for 12 h, tobacco and caffeine for 4 h, and to have fasted from food and alcohol for 6 h prior to assessment (35). All prescribed medications were continued as usual. Following arrival, the participants were familiarized with the lab space, equipment, operators, and research protocols. Height (SECA220 stadiometer, SECA GMBH & Co., Germany) and body composition (InBody 230, Biospace, Seoul, South Korea) were then measured.

#### Pulse Wave Analysis (PWA)

Supine PWA was assessed automatically using the SphygmoCor XCEL System v1 (AtCor Medical Pty Ltd., Sydney, Australia) technology and software (SphygmoCor XCEL Software Version: 1.2). Briefly, a pneumatic blood pressure cuff connected to the SphygmoCor® EXCEL was fitted firmly over the participant's right upper arm and aligned over the brachial artery. The participant then rested for 5-min in a supine position. The Sphygmocor XCEL software was used to initiate the cuff inflation for the automatic PWA measurement. In this assessment, the brachial cuff automatically inflates to measure brachial systolic and diastolic pressure, then it deflates and automatically re-inflates after 5 s to capture the PWA waveform (35). The participant's brachial blood pressure, and AIx measurements were then recorded separately onto an Excel spreadsheet. As heart rate has a profound effect on AIx (36), the AIx was normalized to a heart rate of 75 bpm (AIx75).

#### Pulse Wave Velocity (PWV)

Measurement sites for the PWV analysis were the right carotid artery and right femoral artery (2). The direct method was used to determine the PWV distance as this technique is less complex (less risk of compounding error) than the subtraction method and is more commonly used in arterial stiffness research (17). The location of the strongest carotid pulse was palpated and then marked on the participant's neck. A femoral cuff was then fitted as high up as possible on the participants' right thigh over either thin clothing or bare skin. The distance between the carotid mark and the top edge of the femoral cuff was measured using SECA 207 folding calipers (Hamburg, Germany) designed to increase measurement accuracy in individuals with large breast size or abdominal obesity (2, 37, 38). The distance between the femoral artery and the femoral cuff was then measured by flexing the participant's hip to locate the participant's inguinal crease, and then measuring the distance between the top edge of the femoral cuff and the likely location of the femoral artery in the inguinal crease. The straight line “femoral to cuff” and the “carotid to cuff” distances, were entered into the SphygmoCor software. The “femoral to cuff” measurement is automatically subtracted from the “carotid to cuff” measure to indicate the carotid-femoral distance (typically 500–800 mm) which is the distance used in calculating PWV.

The brachial blood pressure was recorded after the PWA measurement. Then, the capture time (either 5, 10, or 20 s) was selected in the SphygmoCor software. The tonometer was positioned over the marked location with the strongest carotid pulse previously detected by the operator. To standardize recordings and reduce subjectivity, the device was set to automatically capture the pulse wave recording after high quality waveforms had been recorded during the specified capture time (i.e., 5, 10, or 20 s). An “automatic capture” was attempted for a full cuff inflation/deflation cycle, plus the first 20 s of the second cuff inflation. Thereafter, if an automatic capture was unsuccessful but the waveforms appeared to be of sufficient quality to the operator, a manual capture measurement was taken. The operator indicated whether a manual or automatic measurement was taken. Regardless of the automatic or manual capture, the measurement quality was assessed automatically by the SphygmoCor software (based on consistent pulse peaks, troughs and amplitude), and were indicated with either a green “quality controlled” tick, or a red cross indicating a lower quality measure (35). All data for inclusion in this research project were required to have a quality-controlled tick. Any manual capture which did not meet the quality control standards was repeated.

### Statistics

Participants' data were ordered chronologically according to test date and time. Data were then arranged into quintiles to detect any longitudinal differences between the experienced and novice operators.

A simple linear regression was used to determine the validity of the PWA (AIx75) and PWV (m/s) between the experienced (criterion measure) and the novice (practical measure) operators. Measurements were all log transformed prior to analysis, and then back-transformed into a coefficient of variation (CV; Standard deviation expressed as a % of the mean) to reduce non-uniformity of errors (39). Where a dataset had a negative value, a constant (absolute of the maximum negative value +1) was added to all observations in the dataset such that the smallest observation was always 1.

The CV and the validity correlation coefficient (Pearson correlation) were presented to reflect the prediction error, and variable alignment, respectively. The validity correlations were interpreted using the following scale: <0.1, trivial; 0.1–0.3, small; 0.3–0.5, moderate; 0.5–0.7 large; 0.7–0.9, very large; >0.9 extremely large to indicate the level of agreement between the two operators. Correlations >0.9 are considered acceptable for validity studies (39).

Finally, the difference in the experienced and novice operator's measurements was determined, along with the average and SD of these differences for each quintile. The accuracy of the measurement was assessed using both the mean difference and the SD and was interpreted according to the ARTERY Society Guidelines whereby: Excellent: mean difference ≤ 0.5 m/s and SD ≤ 0.8 m/s; acceptable: mean difference <1.0 m/s and SD <1.5 m/s; and poor: mean difference ≥ 1.0 m/s or SD > 1.5 m/s (40).

## Results

**Figure 1:**
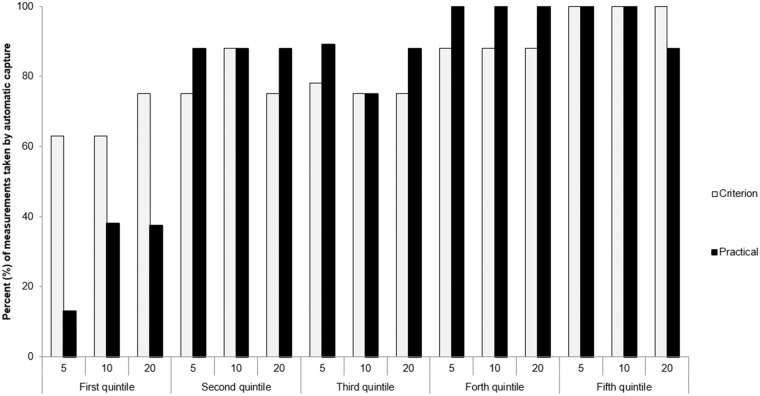
Proportion of “automatic captures” for the experienced (criterion) and novice (practical) operators during the 3 time recording windows (5, 10, 20 s). Quintiles were ordered chronologically according to test date and time (Quintile 1 contains first participants tested).

For PWA, the quintile-derived averages for the AIx75 measurements were closer between novice and experience operators than the AIx measurements (see Table 1). Statistics in Table 1 indicate that all quintile-derived PWV averages across all time-periods were similar between novice and experienced operators. Measurement similarity between averages worsened in quintile 5 in all measurements (Table 1). In all quintiles, the experienced operator had a distinct measurement advantage given this person was much more trained than the novice operator. As a result, there was a positive bias in the mean of the differences between operators as indicated in Figure 1 with all mean differences being plotted above 0.

**Table 1:** Means and standard deviations for PWA and PWV measurements recorded by an experienced (criterion) and novice (practical) operator.

	**Quintile 1** **(*****n*** **=** **8)**		**Quintile 2** **(*****n*** **=** **8)**		**Quintile 3** **(*****n*** **=** 9)^*^		**Quintile 4** **(*****n*** **=** **8)**		**Quintile 5** **(*****n*** **=** **8)**	
	**Criterion**	**Practical**	**Criterion**	**Practical**	**Criterion**	**Practical**	**Criterion**	**Practical**	**Criterion**	**Practical**
**PULSE WAVE ANALYSIS**										
AIx	18.6 ± 14.9	17.8 ± 11.3	15.4 ± 10.1	19.0 ± 11.0	29.4 ± 11.4	29.1 ± 9.1	20.1 ± 8.4	18.9 ± 10.8	20.9 ± 14.3	14.0 ± 14.1
AIx75	10.9 ± 14.0	9.6 ± 9.9	9.5 ± 9.8	8.4 ± 9.7	21.3 ± 13.0	21.9 ± 10.7	11.3 ± 10.0	11.1 ± 12.9	12.0 ± 17.6	4.8 ± 15.9
**PULSE WAVE VELOCITY**										
5 s PWV (m/s)	9.8 ± 1.6	8.5 ± 3.0	9.6 ± 1.12	9.2 ± 0.8	10.1 ± 1.7	10.4 ± 1.8	9.6 ± 1.0	9.3 ± 1.1	9.4 ± 1.2	8.8 ± 1.3
10 s PWV (m/s)	10.1 ± 1.3	9.6 ± 1.6	9.6 ± 1.7	9.4 ± 1.6	10.0 ± 2.1	9.9 ± 1.6	9.5 ± 0.9	9.3 ± 1.2	9.6 ± 1.2	8.7 ± 1.2
20 s PWV (m/s)	10.1 ± 1.4	9.0 ± 2.7	9.8 ± 1.5	9.7 ± 1.6	10.1 ± 1.9	10.0 ± 1.6	9.6 ± 0.9	9.4 ± 1.2	9.4 ±1.3	8.8 ± 1.2

*Data were arranged chronologically based on the date and time of the participant's assessment, and then divided into quintiles in order to assess the progressive effect of learning on measurement accuracy. Data are presented as mean ± standard deviation*.

* *10 and 20 s PWV quintile 3 groups had 8 participants*.

During measurement capture, the novice operator relied on predominantly manual capture measurements in the first quintile. In Quintiles 2–5 both the novice and experienced operators relied predominantly (>75%) on automatically captured measurement (Figure 1).

**Figure 2:**
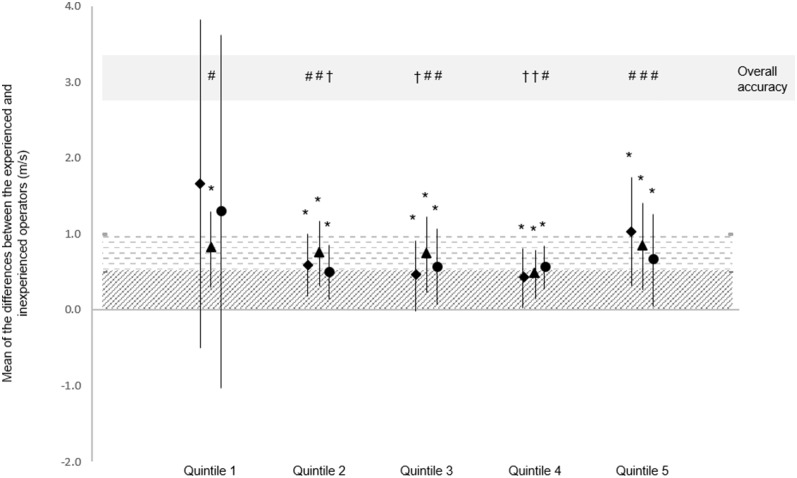
Mean difference and SD of the mean differences between experienced and novice operator, and the qualitative outcome. Data represent the mean of the differences in PWV measured by experienced and novice operators, and the SD of the mean differences. Accuracy of the mean differences is represented using diagonal stripes (excellent accuracy) and horizontal stripes (acceptable accuracy). *Excellent SD accuracy. Overall accuracy is the worse of either the mean difference or SD qualitative interpretation: ^†^Excellent overall accuracy; ^#^Acceptable overall accuracy. ^##^PWV from 5 s recording; ^†^^†^PWV from 10 s recording; ^###^PWV from 20 s recording. Quintiles were ordered chronologically according to test date and time (Quintile 1 contains first participants tested).

On the whole, measurement reliability (intraclass correlation) improved and measurement variability (coefficient of variation) decreased across the first 4 quintiles, particularly between quintiles 1 and 2 (Table 2). Measurement variability between operators in the 5, 10, and 20 s PWV measurement periods all demonstrated “acceptable” agreement from quintile 2 onwards (Figure 2), despite worsening in quintile 5 (Table 2).

**Table 2:** Typical error and validity correlation of the log-transformed data for main measures of PWA and PWV between the experienced (criterion) and novice (practical) operators.

	**Quintile 1**		**Quintile 2**		**Quintile 3**		**Quintile 4**		**Quintile 5**	
	**Validity correlation (90% CL) qualitative interpretation**	**CV (%)**	**Validity correlation (90% CL) qualitative interpretation**	**CV (%)**	**Validity correlation (90% CL) qualitative interpretation**	**CV (%)**	**Validity correlation (90% CL) qualitative interpretation**	**CV (%)**	**Validity correlation (90% CL) qualitative interpretation**	**CV (%)**
5 s PWV	0.5 (−0.2 – 0.9)	16.1 (10.9 – 33.2)	0.8 (0.4 – 0.96)	7.3 (5 – 14.4)	0.95 (0.8 – 0.99)^*^	6.2 (4.4 – 11.5)	0.9 (0.6 – 0.97)	4.8 (3.3 – 9.5)	0.6 (−0.0 – 0.9)	10.5 (7.1 – 21.0)
	Moderate		Very large		Extremely large		Very large		Large	
10 s PWV	0.8 (0.4 – 0.95)	8.2 (5.6 – 16.3)	0.8 (0.4 – 0.96)	10.5 (7.1 – 21.0)	0.9 (0.7 – 0.98)^*^	9.1 (6.2 – 18.1)	0.9 (0.5 – 0.97)	4.7 (3.2 – 9.2)	0.9 (0.6 – 0.97)	6.2 (4.2 – 12.2)
	Very large		Very large		Extremely large		Very large		Very large	
20 s PWV	0.2 (−0.5 – 0.8)	14.8 (10 – 30.2)	0.9 (0.7 – 0.98)^*^	6.9 (4.7 – 13.7)	0.9 (0.7 – 0.98)^*^	8.6 (5.9 – 17.2)	0.8 (0.4 – 0.95)	6 (4.1 – 11.9)	0.9 (0.5 – 0.97)	7.4 (5 – 14.6)
	Small		Extremely large		Extremely large		Very large		Very large	
AIx75	0.7 (0.1 – 0.9)	67.5 (42.8 – 168.5)	0.8 (0.3 – 0.94)	39.3 (25.7 – 88.7)	0.8 (0.4 – 0.9)	25.5 (17.4 – 50.5)	0.97 (0.88 – 0.99)^*^	15 (10.2 – 30.8)	0.97 (0.87 – 0.99)^*^	25.3 (16.9 – 54.1)
	Large		Very large		Very large		Extremely large		Extremely large	

* *r > 0.9; Data were arranged chronologically based on the date and time of the participant's assessment, and then divided into quintiles in order to assess the progressive effect of learning on performance. CL, Confidence limits; qualitative interpretation: the magnitude of the agreement between the novice and experienced operator's measurements; CV, coefficient of variation; 5 s PWV, pulse wave velocity measured over a 5-s window; 10 s PWV, pulse wave velocity measured over a 10-s window; 20 s PWV, pulse wave velocity measured over a 20-s window*.

## Discussion

The current research is the first to examine the variation in PWA and PWV measurements between a novice and experienced operator. Furthermore, this research is the first to report the differences in measurement accuracy between PWV recording lengths of different durations. Our study found that measurement agreement and accuracy substantially improved between Quintiles 1 and 2 and was typically maintained or improved (with small variability) from quintiles 2–4. These findings indicate a very steep initial learning curve (over quintile 1 + 5 pre-study practice measurements), where after small variations in agreement between experienced and novice operators occurred. The measurement accuracy was similar between 5, 10, and 20 s recording periods in quintiles 2–5.

In the last quintile, the agreement for both AIx75 and PWV worsened. It is possible that over the course of the intensive 2-week data-collection period, which started at 5:30 a.m. and typically persisted for 6 h, that the operators were demonstrating signs of mental fatigue. The data collected over a 2-week period was evenly spread with a minimum of 3 and a maximum of 6 participants measured per morning, accounting for ~20 participants per week. Monday was a practice testing day where 3 of the 5 training participants were tested as a way to run through the official testing protocol with the novice and expert operators measuring in random order. Tuesday marked the first day of data collection which would be used for analysis and this lasted for two consecutive work weeks with the most popular day to test being Thursday. In order to minimize operator fatigue and measurement errors, the maximum number of participants tested was kept to 6 per morning. Tuesdays and Fridays had 3–4 participants where Monday, Wednesday, and Thursday had 5–6 participants. Both operators found that an even spread of 3–6 participants per morning allowed for learning through training repetition without inducing too much measurement fatigue. Some of the common effects of mental fatigue include changes in mood, task motivation, and performance deficit (41). As the effects of mental fatigue may be more pronounced in unfamiliar tasks vs. automatic tasks [as cited by van der Linden et al., Broadbent and Broadbent, and Hockey (41–43)], the accumulating increase in mental fatigue may have had a greater impact on the novice operator compared to the experienced operator. The imbalance in this effect may be responsible for the increase in the measurement variability in quintile 5.

Alternatively, personal factors such as overconfidence, or, more specifically, overestimation of one's actual performance (44) may have reduced measurement agreement in the last quintile. Overestimation is often associated with a second form of overconfidence known as overplacement, wherein a person believes their performance to be better in relation to others (44). Interestingly, some research has indicated that an increase in personal familiarity without an increase in observing others in a similar context increases overplacement (44). However, overestimation is more likely to arise from imbalanced information about one's own performance compared to others (specifically that one has worse knowledge of others than of oneself) (44) than of self-seeking or wishful thinking (45). Unfortunately, overconfidence can result in poorer decision-making and poorer outcomes (45) than if better self-regulation and monitoring strategies are employed (46). In the present study, the novice operator gained familiarity with the equipment over the course of the study, but, to avoid research bias, was not privy to the experienced researcher's measurement sessions. This research design, while scientifically thorough, possibly contributed to overconfidence and, ironically, to poorer learning outcomes on the part of the novice operator.

### PWV Comparison With Other Studies

Our findings support those of Grillo and colleagues who suggested that acceptable—excellent measurement recordings require a relatively short learning period (47). Their CV using the SphygmoCor Vx in patients with high cardiovascular risk was higher (CV = 9.5%) than the CV in the present study after a similar amount of training (2 weeks). The SphygmoCor XCEL intra-test reliability reported by Hwang et al. (26) was considerably smaller than our study (stronger validity correlation: *r* > 0.99 vs. *r* ~ 0.9 in our study) however, no CV of the intra-test measurement differences were presented. The closer measurement repeatability in Hwang et al.'s (26) study is possibly due to a single, well-experienced researcher taking all measurements.

The inter-operator PWV agreement in quintiles 2–4 in our study support previous research. For example, our levels of agreement were slightly higher than those reported by experienced operators measuring PWV in patients with kidney failure (48). That is, Frimodt-Møller et al. (48) reported a mean inter-operator difference in aortic PWV of 0.3 m/s compared to a range of 1–4 m/s in quintiles 2–4 our study. However, our study reported better levels of agreement when compared to Grillo et al. (47) who assessed PWV in patients with high cardiovascular risk. Even when Grillo et al. (47) analyzed inter-operator variability in a sub-set of patients with reasonable arterial compliance (i.e., a PWV of <10 m/s), our CV typically remained lower than their data (PWV < 10 m/s = CV of 8.5% vs. PWV > 10m/s = CV of 10.4%; compared to a CV range of 4.7–10.5 in quintiles 2–5 in our study).

The participants in our study were middle-aged, mostly physically active, and boarderline normal/overweight, and with generally healthy blood pressure. Therefore, we would have anticipated that the mean PWV values would lie closer to the reference value of 7.2 m/s reported by Mattace-Raso et al. (17). Instead the PWV in our population was only slightly lower than the mean PWV of older patients with kidney disease [PWV ~ 10.5 m/s (47)], and similar to an older population with chronic kidney disease [PWV = 9.9 m/s (48)]. While both Grillo et al. (47) and Frimodt-Møller et al. (48) used Sphygmocor devices to monitor the aortic (carotid-femoral) PWV, their devices were less automated earlier models than the device used in the present study. As different technologies are known to produce different measurements and variances (47), some of the differences seen between studies may reflect equipment discrepancies.

### PWV and Recording Window

The SphygmoCor XCEL device offers three different measurement durations for the capture of PWV waveforms (5, 10, or 20 s). The operator manual indicates that while 5 s is the default setting, longer recording times may be required for participants with slower respiratory cycles and/or with more variable heart rates (31). The “capture time” is not always reported in journal articles and little is known about the differences in measurement agreement or variability between these time selections. Our study found that inter-operator agreement improved considerably in all measurement periods between quintile 1 and 2, suggesting that there is a strong initial learning effect regardless of automatic capture time. The 10-s time-period was the only measurement that yielded acceptable agreement between the novice and experienced researchers in quintile 1 and so may be preferable for training operators. By quintile 4, both the 5- and 10- s recordings had “excellent” acceptibility, very large correlation coefficients and a CV <5%. However, the 5-s measurement was most succeptible to inaccuracy in quintiles 1 and 5 which may indicate that this recording window may be inappropriate for novice operators, or during times where mental fatigue (41) or overestimation (44) may be exhibited.

### PWA Comparison to Other Studies

Our measures of AIx75 in a reasonably healthy population were similar to those reported in other healthy poplations, including the “healthy participants” [AIx = 20.2 (26)], and pregnant females [AIx75 = 11.7 (29)]. Conversely, our data were considerably lower than those taken in ambulatory hospital patients [AIx75 ~ 19 (27)], or patients with high cardiovascular risk [AIx75= 26.6 (49)].

The AIx or AIx75 measurement appears to be a measurement with considerably higher variation than any of the PWV measures. This has also been reported in other studies with Magda et al. (49) reporting excellent PWV inter-operator variability of 2.5% but only satisfactory inter-operator variability of 8.4% for AIx. Indeed, Magda et al. reported an even higher intra-operator difference of 17.8% for AIx which further demonstrates the varaibility of this measure. The CVs reported by Magda et al. (49) were about half the size of the present study. These could have been attributed to the longer experience (~35 practice measurments, and a 2-day training workshop) of the data collectors in their study, or to the differences in the Complior and SphygmoCor devices.

The health of the participants my also influence the level of inter-operator AIx75 agreement. Our study (quintiles 2–4) reported slightly better mean inter-operator differences in AIx75 than studies using kidney disease patients (48) or ambulatory hospital patients (27). On the other hand, our study reports ICCs for AIx that are similar to those of Hwang et al. (26) in their population of healthy adults of a similar age to our participants. In particular, Hwang and colleagues reported similar ICCs (*r* = 0.98) to ours (*r* = 0.97 in quintiles 4 and 5), as well as reporting similar mean differences over consecutive measurements (AIx range: ~1.1 in Hwang et al.'s study which was similar to the mean difference range in quintiles 3 and 4 in our study).

Despite excellent coefficients of variation and small differences in mean, the CV associated with AIx are reasonably large (15–25% in the last 3 quintiles in our study). However, when the average AIx75 measurements taken in triplicate was used, the trained nurses (2-day workshop and ~35 practice measurements) demonstrated an inter-technican AIx difference of 0.1 (29). Therefore, an average of 2–3 AIx measurements may be more reliable than taking only one measurement.

There is little research available regarding potential reasons for the considerably higher variability in AIx. The AIx is a complex measurement that requires accurate capture of the pulse waveform including accurate measurement of the first and second systolic peaks as well as the pulse pressure (2). As our arteries are dynamic organs which are continually responding to alterations in shear stress (50, 51) through both flow-mediated dilation and constriction (50), the participant's internal and external environments may have transient effects on the augmentation index. This measurement complexity combined with the automatic nature of the measurement could be responsible for higher variability. That is, while using the tonometer to assess PWV, the operator may make slight adjustments to the tonometer placement or pressure in order to maximize the quality of the waveform capture. However, once the cuff has been fastened to the participant's upper arm in the assessment of AIx, the operator has very little control over the quality of the measurement as it is no longer possible to adjust or correct cuff pressure or placement in response to subtle changes in physiological landmarks or for any external stimuli that may interefere with the measurement. However, with greater practice, cuff placement and fit may become more consistent, and instructions to the particpant may become clearer, all resulting in a more accurate measurement. Unfortunately there is no research available to support or refute this suggestion and further work in this area is required.

### Automatic Capture Findings

Recordings using the equipment's “automatic capture” function was prioritized over manual measurements to standardize outcomes. To this end, the novice operator depended primarily on the manual capture technique in the first quintile (*n* = 8), but by the second quintile demonstrated similar automatic capture proportions to the experienced operator (Figure 1). There was also a gradual increase in the proportion of the experienced operators' “automatic capture” measurements from the first to the fifth quintile. These data suggest that experienced operators may also benefit from practice measurements prior to a large research study.

### Limitations

The limitations associated with reliability and validity studies is the manner in which the levels of agreement have been interpreted. While we have elected to examine variability between researchers using the ICC and CV, others have preferred to use Bland-Altman plots. Generally the studies using Bland-Altman plots have reported high PWA reproducibility (26, 27, 29, 48). However, our interpretation of reliability using the CV to represent the typical error of the estimate or prediction error, and the ICC to link the assessments (52), appears to be more conservative particularly regarding the AIx75 outcomes. These differences in methodological approaches makes comparisons between studies more challenging. In the end, we attempted to compare our reliability with others using primarily the inter-operator difference in means, which does not account for any variation in the measurements and therefore can only provide limited substance to our comparison.

Another limitation is that participant numbers in each of the quintiles (*n* = 8) was small, and therefore may reduce the statistical power of the analyses. A power calculation was made a priori. The sample size needed in this inter-operator reliability study with two operators was designed to achieve a kappa value of K = 0.80, with an assumed probability of positive ratings of 0.30, and a desired width of the CI at *w* = 0.20, with a 0.95 level of confidence as suggested by Shoukri et al. (53) equates to a total sample size of 117 instead of the 41 recruited. The sample was limited as the study took place at a university which was nearing a major holiday so fewer participants were available than anticipated. However, having smaller groups does provide better insight into the individual variation for each participant, which is something that is important in a practical or clinical context. Furthermore, some data was necessarily excluded from the dataset due to equipment failure or major errors in technique related to the learning process but rendering the data inaccurate.

Moreover, the participant cohort studied was atypical for the majority of research studies and patient work conducted in clinical settings, as they were healthy (free from cardiovascular disease) and generally physically fit (78% physically active). Therefore, training recommendations in this study are merely the minimum recommendations and might need to be higher for novice operators aiming to accurately record PWV from unfit and unhealthy populations, i.e., with cardiovascular diseases.

As there was only one novice researcher being compared against one experienced researcher, considerable variation might exist between the findings of this study and other studies involving different experience gaps between operators or between other novice operators. For example, the swiftness of the learning response will vary depending on the ability of the operator to learn and understand a new skill (using a tonometer) as well as the ability to keep a steady hand, locate the anatomical landmarks, detect regions of the strongest pulse, measure distances accurately, and react quickly sensitively to physiological changes to capture good data. Future research should consider comparing an experienced operator with a larger number of novice operators.

Finally, as there is only one study which used the SphygmoCor XCEL device (26), we have compared our findings with other studies using different techniques and devices which may add further differences between outcomes. Moreover, this study examines a participant cohort that may vary for what is considered typical in research or clinical work.

### Recommendations and Conclusion

Our study reported acceptable—excellent PWV measurement accuracy by a novice operator following as little as 14 practice participants (5 practice participants + 8 participants in quintile 1 and one extra for tonometer measurement difficulty). Counter-intuitively, the automatic AIx75 measurement required more practice before measurement agreement reached acceptable levels of *r* > 0.9 (39). For both AIx75 and PWV, measurement accuracy typically continued to improve over the first 4 quintiles (increased validity correlation and reduced CV). Therefore, for operators who have no experience measuring PWV with the SphygmoCor XCEL, we recommend a practice period of at least 14 participants prior to practical data collection (Figure 2), and ideally 30 participants (5 practice participants + first 3 quintiles which yielded excellent overall accuracy for 5 and 10 s time-periods, and borderline-excellent overall accuracy for the 20 s time-period, Figure 2) for either research or clinical use. The 10 s measurement capture interval appeared to provide the most accurate measurements for beginner users.

Despite AIx75 being an automated measurement using a pneumatic brachial cuff, the measurement itself is more complex (involving numerous variables detected from a pressure waveform). As such the AIx is associated with a considerably higher inter-operator CV. One critical finding that should be strongly considered for future implications is that both operators experienced a general inability to capture PWV data from one participant who had a seemingly high proportion of neck adipose tissue, making the carotid pulse difficult to detect with the tonometer. Since it is impossible to predict the likelihood of measuring someone with high neck adipose tissue, we advise testing one extra person (*n* = 14) during the PWV training due to potential measurement difficulties but the AIx was unaffected by such a case so it remains unchanged at a recommended minimum of 30 training participants (5 practice measurements + first 3 quintiles for *r* >0.9, and lowest CV of 15%) prior to clinical data collection.

Mental fatigue, and/or over-confidence may play a role in measurement accuracy, particularly in prolonged measurement intervals. Therefore, we recommend that operators are well-rested at the time of data capture, that the intensity of the measurement periods is moderated by taking regular breaks. Collaboration with experts/support throughout the learning process should be available to encourage a meticulous and reflective attitude to data collection.

## Data Availability Statement

The datasets generated for this study are available on request to the corresponding author.

## Ethics Statement

The studies involving human participants were reviewed and approved by Lincoln University Human Ethics Committee. The patients/participants provided their written informed consent to participate in this study.

## Author Contributions

CE and MH designed and planned the study. MH wrote and submitted the ethical application. CE and CL contributed to participant recruitment, data collection, and data entry. MH and CL conducted data analysis. MH, CE, and CL contributed to drafting different sections of the manuscript. All authors finalized the draft and CE submitted the final manuscript.

## Conflict of Interest

The authors declare that the research was conducted in the absence of any commercial or financial relationships that could be construed as a potential conflict of interest. The handling editor declared a past co-authorship with one of the authors MH.

## References

[1] Dumor K Shoemaker-Moyle M Nistala R Whaley-Connell A. Arterial stiffness in hypertension: an update. Curr Hypertens Rep. (2018) 20:1–8. 10.1007/s11906-018-0867-x 29974262

[2] Laurent S Cockcroft J van Bortel L Boutouyrie P Giannattasio C Hayoz D Abridged version of the expert consensus document on arterial stiffness. Artery Res. (2007) 1:2–12. 10.1016/j.artres.2007.03.003 17000623

[3] Sakuragi S Abhayaratna WP. Arterial stiffness: methods of measurement, physiologic determinants and prediction of cardiovascular outcomes. Int J Cardiol. (2010) 138:112–8. 10.1016/j.ijcard.2009.04.027 19473713

[4] Ben-Shlomo Y Spears M Boustred C May M Anderson SG Benjamin EJ. Aortic pulse wave velocity improves cardiovascular event prediction: an individual participant meta-analysis of prospective observational data from 17,635 subjects. J Am Coll Cardiol. (2014) 63:636–46. 10.1016/j.jacc.2013.09.063 24239664 PMC4401072

[5] Lizamore CA Stoner L Lucas SJE Lucero A Hamlin MJ. Does arterial health affect VO2peak and muscle oxygenation in a sedentary cohort? Med Sci Sports Exerc. (2015) 47:272–9. 10.1249/MSS.0000000000000414 24983339

[6] Coutinho T Turner ST Kullo IJ. Aortic pulse wave velocity is associated with measures of subclinical target organ damage. JACC Cardiovasc Imaging. (2011) 4:754–61. 10.1016/j.jcmg.2011.04.011 21757166 PMC3862768

[7] Abbatecola AM Chiodini P Gallo C Lakatta E Sutton-Tyrrell K Tylavsky FA. Pulse wave velocity is associated with muscle mass decline: health ABC study. AGE. (2012) 34:469–78. 10.1007/s11357-011-9238-0 21479573 PMC3312626

[8] Rodríguez AJ Karim MN Srikanth V Ebeling PR Scott D. Lower muscle tissue is associated with higher pulse wave velocity: a systematic review and meta-analysis of observational study data. Clin Exp Pharmacol Physiol. (2017) 44:980–92. 10.1111/1440-1681.12805 28656698

[9] Angermann S Baumann M Wassertheurer S Mayer CC Steubl D Hauser C. Pulse wave velocity is associated with cognitive impairment in hemodialysis patients. Clin Sci. (2017) 131:1483–93. 10.1042/CS20170087 28495909

[10] Elias MF Robbins MA Budge MM Abhayaratna WP Dore GA Elias PK. Arterial pulse wave velocity and cognition with advancing age. Hypertension. (2009) 53:668–73. 10.1161/HYPERTENSIONAHA.108.126342 19237680 PMC2716128

[11] Chen S Chetty S Lowenthal A Evans JM Vu C Stauffer KJ. Feasibility of neonatal pulse wave velocity and association with maternal hemoglobin A1c. Neonatology. (2015) 107:20–6. 10.1159/000366467 25301402

[12] Hudson LD Rapala A Khan T Williams B Viner RM. Evidence for contemporary arterial stiffening in obese children and adolescents using pulse wave velocity: a systematic review and meta-analysis. Atherosclerosis. (2015) 241:376–86. 10.1016/j.atherosclerosis.2015.05.014 26071661

[13] Stoner L Lambrick DM Westrupp N Young J Faulkner J. Validation of oscillometric pulse wave analysis measurements in children. Am J Hypertens. (2014) 27:865–72. 10.1093/ajh/hpt243 24390294

[14] Huang C Wang J Deng S She Q Wu L. The effects of aerobic endurance exercise on pulse wave velocity and intima media thickness in adults: a systematic review and meta-analysis. Scand J Med Sci Sports. (2016) 26:478–87. 10.1111/sms.12495 26059748

[15] El-Chilali K Farouk H Abdelhafez M Neumann T Alotaibi S Wendt D. Predictors of aortic pulse wave velocity in the elderly with severe aortic stenosis. Aging Clin Exp Res. (2016) 28:519–25. 10.1007/s40520-015-0443-z 26349567

[16] Colin E Arbez L Mourot L Laurant P Tordi N. Late effects of cycle competition on arterial stiffness: a preliminary study. J Sports Med Phys Fitness. (2006) 46:116–21. 16596109

[17] Mattace-Raso FUS Hofman A Verwoert GC Witteman JCM Wilkinson I Cockcroft J Determinants of pulse wave velocity in healthy people and in the presence of cardiovascular risk factors: ‘establishing normal and reference values'. Eur Heart J. (2010) 31:2338–50. 10.1093/eurheartj/ehq165 20530030 PMC2948201

[18] Choi J Kim S-Y Joo S-J Kim K-S. Augmentation index is associated with coronary revascularization in patients with high Framingham risk scores: a hospital-based observational study. BMC Cardiovasc Disord. (2015) 15:131. 10.1186/s12872-015-0123-0 26481213 PMC4615329

[19] Xaplanteris P Vlachopoulos C Vyssoulis G Dima I Terentes-Printzios D Ioakeimidis N Pulse wave velocity and augmentation index are associated with 10-year general cardiovascular risk and heart/vascular age in newly diagnosed, never-treated hypertension. Artery Res. (2009) 3:182 10.1016/j.artres.2009.10.039

[20] Binder J Bailey KR Seward JB Squires RW Kunihiro T Hensrud DD. Aortic augmentation index is inversely associated with cardiorespiratory fitness in men without known coronary heart disease. Am J Hypertens. (2006) 19:1019–24. 10.1016/j.amjhyper.2006.02.012 17027821

[21] Borresen ML Wang D Strandgaard S. Pulse wave reflection is amplified in normotensive patients with autosomal-dominant polycystic kidney disease and normal renal function. Am J Nephrol. (2007) 27:240–6. 10.1159/000101369 17389784

[22] Kalil GZ Kalil GZ Recober A Hoang-Tienor A Bridget Zimmerman M. Higher augmentation index is associated with tension-type headache and migraine in middle-aged/older humans with obesity. Obesity. (2016) 24:865–70. 10.1002/oby.21414 26847595 PMC5539769

[23] Vogrin B Slak Rupnik M Mičetić-Turk D. Increased augmentation index and central systolic arterial pressure are associated with lower school and motor performance in young adolescents. J Int Med Res. (2017) 45:1892–900. 10.1177/0300060516678717 28703627 PMC5805191

[24] Butlin M Qasem A. Large artery stiffness assessment using SphygmoCor technology. Pulse. (2017) 4:180–92. 10.1159/000452448 28229053 PMC5290450

[25] Butlin M Qasem A Battista F Bozec E McEniery CM Millet-Amaury E. Carotid-femoral pulse wave velocity assessment using novel cuff-based techniques: comparison with tonometric measurement. J Hypertens. (2013) 31:2237–43. 10.1097/HJH.0b013e328363c789 24077246

[26] Hwang MH Yoo JK Kim HK Hwang CL Mackay K Hemstreet O. Validity and reliability of aortic pulse wave velocity and augmentation index determined by the new cuff-based SphygmoCor Xcel. J Hum Hypertens. (2014) 28:475–81. 10.1038/jhh.2013.144 24430704

[27] Crilly M Coch C Bruce M Clark H Williams D. Indices of cardiovascular function derived from peripheral pulse wave analysis using radial applanation tonometry: a measurement repeatability study. Vascular Med. (2007) 12:189–97. 10.1177/1358863X07081134 17848475

[28] McGreevy C Barry M Bennett K Williams D. Repeatability of the measurement of aortic pulse wave velocity (aPWV) in the clinical assessment of arterial stiffness in community-dwelling older patients using the Vicorder® device. Scand J Clin Lab Invest. (2013) 73:269–73. 10.3109/00365513.2013.770162 23544457

[29] Crilly MA Orme KM Henderson J Allan AJ Bhattacharya S. Repeatability of SphygmoCor pulse wave analysis in assessing arterial wave reflection in pregnancy using applanation tonometry. Hypertens Pregnancy. (2014) 33:322–32. 10.3109/10641955.2013.877926 24475771

[30] Vlachopoulos C Aznaouridis K Stefanadis C. Prediction of cardiovascular events and all-cause mortality with arterial stiffness: a systematic review and meta-analysis. J Am Coll Cardiol. (2010) 55:1318–27. 10.1016/j.jacc.2009.10.061 20338492

[31] SphygmoCor XCEL Operator's Manual. SphygmoCor XCEL System v1. Sydney, NSW: AtCor Medical Pty. Ltd (2016).

[32] O'Rourke MF. Carotid artery tonometry: pros and cons. Am J Hypertens. (2015) 29:296–8. 10.1093/ajh/hpv194 26687920

[33] Wilkinson IB Cockcroft JR Webb DJ. Pulse wave analysis and arterial stiffness. J Cardiovasc Pharmacol. (1998) 32:S33–S7. 9883745

[34] Segers P Kips J Trachet B Swillens A Vermeersch S Mahieu D Limitations and pitfalls of non-invasive measurement of arterial pressure wave reflections and pulse wave velocity. Artery Res. (2009) 3:79–88. 10.1016/j.artres.2009.02.006

[35] AtCor Medical Pty. Ltd Operator's manual sphygmoCor XCEL system v1. In: XCEL S, editor. SphygmoCor XCEL: Simply the Gold Standard. Sydney, NSW: AtCor Medical Pty. Ltd (2012). p. 99.

[36] Wilkinson IB MacCallum H Flint L Cockcroft JR Newby DE Webb DJ. The influence of heart rate on augmentation index and central arterial pressure in humans. J Physiol. (2000) 525:263–70. 10.1111/j.1469-7793.2000.t01-1-00263.x 10811742 PMC2269933

[37] Rajzer MW Wojciechowska W Klocek M Palka I Brzozowska-Kiszka M Kawecka-Jaszcz K. Comparison of aortic pulse wave velocity measured by three techniques: Complior, SphygmoCor and Arteriograph. J Hypertens. (2008) 26:2001–7. 10.1097/HJH.0b013e32830a4a25 18806624

[38] Weber T Ammer M Rammer M Adji A O'Rourke MF Wassertheurer S. Noninvasive determination of carotid-femoral pulse wave velocity depends critically on assessment of travel distance: a comparison with invasive measurement. J Hypertens. (2009) 27:1624–30. 10.1097/HJH.0b013e32832cb04e 19531964

[39] Hopkins W Linear models and effect magnitudes for reserach, clinical and practical applications. Sport Sci. (2010) 14:49–57.

[40] Wilkinson IB McEniery CM Schillaci G Boutouyrie P Segers P Donald A ARTERY Society guidelines for validation of non-invasive haemodynamic measurement devices: part 1, arterial pulse wave velocity. Artery Res. (2010) 4:34–40. 10.1016/j.artres.2010.03.001

[41] van der Linden D Frese M Meijman TF. Mental fatigue and the control of cognitive processes: effects on perseveration and planning. Acta Psychol. (2003) 113:45–65. 10.1016/S0001-6918(02)00150-6 12679043

[42] Broadbent DE Broadbent DE. Is a fatigue test now possible? Ergonomics. (1979) 22:1277–90. 10.1080/00140137908924702 540644

[43] Hockey GRJ Cognitive-energetical control mechanisms in the management of work demands and psychological health. In: Baddeley AD, Weiskrantz L, editors. Attention: Selection, awareness, and control: A tribute to Donald Broadbent. New York, NY: Clarendon Press; Oxford University Press (1993). p. 328–45.

[44] Moore DA Healy PJ. The trouble with overconfidence. Psychol Rev. (2008) 115:502–17. 10.1037/0033-295X.115.2.502 18426301

[45] Moore DA Moore DA Schatz D The three faces Of overconfidence. Soc Pers Psychol Compass. (2017) 11:e12331 10.1111/spc3.12331

[46] de Bruin ABH de Bruin ABH Kok EM Lobbestael J de Grip A The impact of an online tool for monitoring and regulating learning at university: overconfidence, learning strategy, and personality. Metacogn Learn. (2017) 12:21–43. 10.1007/s11409-016-9159-5

[47] Grillo A Parati G Rovina M Moretti F Salvi L Gao L. Short-term repeatability of noninvasive aortic pulse wave velocity assessment: comparison between methods and devices. Am J Hypertens. (2017) 31:80–8. 10.1093/ajh/hpx140 29059329

[48] Frimodt-Møller M Nielsen AH Kamper A-L Strandgaard S. Reproducibility of pulse-wave analysis and pulse-wave velocity determination in chronic kidney disease. Nephrol Dial Transplant. (2008) 23:594–600. 10.1093/ndt/gfm470 17989106

[49] Magda SL Ciobanu AO Florescu M Vinereanu D. Comparative reproducibility of the noninvasive ultrasound methods for the assessment of vascular function. Heart Vessels. (2013) 28:143–50. 10.1007/s00380-011-0225-2 22241737

[50] Levenson J Pessana F Gariepy J Armentano R Simon A. Gender differences in wall shear–mediated brachial artery vasoconstriction and vasodilation. J Am Coll Cardiol. (2001) 38:1668–74. 10.1016/S0735-1097(01)01604-7 11704379

[51] Stout M. Flow-mediated dilatation: a review of techniques and applications. Echocardiography. (2009) 26:832–41. 10.1111/j.1540-8175.2009.00927.x 20003021

[52] Hopkins WG Spreadsheets for analysis of validity and reliability. Sport Sci. (2015) 19:36–42.

[53] Shoukri MM Asyali MH Donner A Sample size requirements for the design of reliability study: review and new results. Stat Methods Med Res. (2004) 13:251–71. 10.1191/0962280204sm365ra

